# Development and internal validation of a nomogram to predict perioperative hypothermia in patients undergoing laparoscopic gynecologic surgery under general anesthesia: a retrospective cohort study

**DOI:** 10.3389/fmed.2026.1799286

**Published:** 2026-06-10

**Authors:** Yingbo Ren, Lei Huang, Ling Chen, Feng Liu, Lihong Song, Qingyuan Li, Xin Wang, Hongyan Li

**Affiliations:** 1 Department of Anesthesiology, The Affiliated Traditional Chinese Medicine Hospital, Southwest Medical University, Luzhou, Sichuan, China; 2 Neurology Department, Chengdu Fifth People’s Hospital, The Second Clinical Medical College, Affiliated Fifth People's Hospital of Chengdu University of Traditional Chinese Medicine, Chengdu, Sichuan, China; 3 Department of General Surgery (Vascular Surgery), The Affiliated Hospital, Southwest Medical University, Luzhou, China; 4 Clinical Medical College, Southwest Medical University, Luzhou, Sichuan, China; 5 Chengdu School of Clinical Medicine, Chengdu University of Traditional Chinese Medicine, Chengdu, China

**Keywords:** general anesthesia, laparoscopic gynecologic surgery, nomogram, perioperative hypothermia, risk prediction

## Abstract

**Background:**

Perioperative hypothermia remains a common complication among patients undergoing laparoscopic gynecologic surgery under general anesthesia and is associated with adverse postoperative outcomes. Early identification of patients estimated to be at high risk may facilitate targeted preventive strategies. However, practical and individualized prediction tools for perioperative hypothermia in laparoscopic gynecologic surgery remain limited.

**Methods:**

This single-center, retrospective observational cohort study included adult patients who underwent laparoscopic gynecologic surgery under general anesthesia between January 2019 and February 2024. The prediction model was developed and internally validated using a split-sample design within this gynecologic laparoscopic surgery cohort. Perioperative hypothermia was defined as a core body temperature <36.0 °C during the intraoperative or immediate postoperative period. Patients were randomly divided into a training cohort for model development (*n* = 294) and a split-sample internal validation cohort for model assessment (*n* = 130). Candidate predictors were selected *a priori* based on clinical relevance. Feature selection was performed using least absolute shrinkage and selection operator regression and the Boruta algorithm. A multivariable logistic regression model was developed and visualized as a nomogram. Model discrimination, calibration, and clinical utility were evaluated using the area under the receiver operating characteristic curve (AUC), calibration plots, and decision curve analysis, respectively. Split-sample validation was supplemented by bootstrap optimism-corrected internal validation using 500 resamples.

**Results:**

A total of 424 patients were included, of whom 279 (65.8%) developed perioperative hypothermia, a relatively high incidence that may reflect the outcome definition, routine temperature surveillance, and the characteristics of laparoscopic gynecologic surgery. Feature selection consistently retained age, body mass index, and operative time as the final model predictors. These variables were incorporated into the final prediction model and nomogram. In the training cohort, the model demonstrated good discrimination with an AUC of 0.788 (95% CI, 0.732–0.843), good calibration, and favorable clinical utility. In the split-sample internal validation cohort, discrimination remained stable with an AUC of 0.810 (95% CI, 0.727–0.893), and calibration and decision curve analyses showed consistent model performance. Bootstrap internal validation yielded an optimism-corrected AUC of 0.776 and an optimism-corrected calibration slope of 0.91. At the training-derived Youden cutoff, the model achieved a sensitivity of 0.623 and specificity of 0.853 in the training cohort, and a sensitivity of 0.925 and specificity of 0.580 in the validation cohort.

**Conclusion:**

A simple nomogram based on age, body mass index, and operative time was developed and internally validated to estimate the predicted probability of perioperative hypothermia specifically in adult patients undergoing laparoscopic gynecologic surgery under general anesthesia. Within this surgical setting, the tool may assist clinicians in perioperative risk stratification, triage for intensified temperature monitoring or active warming, and individualized temperature-management planning. External validation in independent laparoscopic gynecologic cohorts is required before broader clinical implementation.

## Introduction

In adult patients undergoing laparoscopic gynecologic surgery under general anesthesia, perioperative hypothermia, commonly defined as a core body temperature below 36.0 °C during surgery or in the immediate postoperative period, remains a clinically relevant complication despite advances in perioperative care (1, 2). The incidence of inadvertent perioperative hypothermia has been reported to range from 30% to over 70%, depending on patient characteristics, surgical procedures, and perioperative temperature management practices (3, 4). Hypothermia is not merely a physiological disturbance; it has been consistently associated with a range of adverse postoperative outcomes, including surgical site infections, coagulopathy, higher transfusion requirements, delayed anesthetic recovery, and prolonged hospital stay (5, 6). Patients undergoing laparoscopic gynecologic surgery under general anesthesia represent a clinically relevant population for hypothermia risk estimation. Although laparoscopic techniques are often perceived as less invasive than open surgery, laparoscopic gynecologic procedures have several setting-specific features that may be associated with perioperative heat loss and heterogeneity in hypothermia occurrence. These features include Trendelenburg positioning, pelvic exposure, prolonged pneumoperitoneum, insufflation of carbon dioxide, extensive pelvic irrigation in selected procedures, variable operative duration, and a relatively high proportion of oncologic operations requiring more complex pelvic dissection (7). In addition, general anesthesia blunts behavioral and autonomic thermoregulation, further increasing susceptibility to hypothermia even in controlled surgical environments (8). These characteristics suggest that prediction models developed in other laparoscopic or non-gynecologic surgical populations may not be directly transferable to laparoscopic gynecologic surgery, supporting the need for a dedicated, setting-specific prediction tool.

Current strategies to prevent perioperative hypothermia primarily focus on universal or protocol-driven warming measures, including forced-air warming, warmed intravenous fluids, and maintenance of operating room temperature (9, 10). While these approaches are effective at a population level, their implementation in routine clinical practice is often inconsistent, and resource limitations may preclude universal application to all patients (11). Importantly, not all patients share the same baseline probability of hypothermia. Patient-related factors, such as advanced age and low body mass index, surgical characteristics, including operative duration, and perioperative management variables may be jointly associated with individual differences in hypothermia occurrence (12, 13). Therefore, early identification of patients estimated to be at high risk within laparoscopic gynecologic surgery may enable more targeted temperature monitoring and warming strategies rather than relying solely on uniform interventions.

Recent studies have increasingly attempted to develop prediction models or nomograms for perioperative or intraoperative hypothermia across different surgical settings, including robotic surgery, gynecological surgery, video-assisted thoracoscopic surgery, general laparoscopic surgery, and gynecological laparoscopic surgery (14, 15). A recent systematic review further summarized the growing body of prediction models for perioperative hypothermia and highlighted the importance of transparent reporting, calibration assessment, and validation (16). These studies provide important evidence for risk stratification, but their clinical applicability may vary according to surgical population, timing of predictor assessment, predictor availability, model complexity, and validation strategy. For laparoscopic gynecologic surgery, procedure-specific factors such as pelvic positioning, pneumoperitoneum duration, irrigation practices, and oncologic surgical complexity may affect the relevance and transportability of predictors selected in other settings. Therefore, additional setting-specific models may still be useful when they are designed for clearly defined surgical populations, rely on routinely available variables, and are reported according to contemporary prediction-modeling standards (17, 18).

Nomograms derived from multivariable prediction models have gained increasing attention in perioperative and anesthesiology research as practical tools for individualized risk estimation (19, 20). By integrating multiple predictors into a single graphical interface, nomograms allow clinicians to estimate outcome probabilities at the bedside and support shared decision-making. Within this evolving literature, the present study was designed to provide a complementary, parsimonious nomogram for patients undergoing laparoscopic gynecologic surgery under general anesthesia. Specifically, we focused on a clearly defined gynecologic laparoscopic cohort, used routinely available demographic and perioperative variables, combined LASSO regression with the Boruta algorithm for feature selection, and evaluated discrimination, calibration, and clinical utility in a split-sample internal validation framework.

Therefore, the present study aimed to develop and internally validate a clinically applicable prediction model for perioperative hypothermia in adult patients undergoing laparoscopic gynecologic surgery under general anesthesia. Using a retrospective cohort design, we applied both least absolute shrinkage and selection operator (LASSO) regression and the Boruta algorithm to retain robust model predictors, constructed a multivariable logistic regression–based nomogram, and evaluated its discrimination, calibration, and clinical utility in training and split-sample internal validation cohorts.

The intended use of the nomogram is to support preoperative or early intraoperative risk stratification, assist triage for intensified temperature monitoring or active warming, and provide a simple risk-estimation tool for perioperative quality-improvement audits within laparoscopic gynecologic surgery.

## Methods

### Study design

This study was designed as a single-center, retrospective observational cohort study aimed at developing and internally validating a prediction model for perioperative hypothermia in patients undergoing laparoscopic gynecologic surgery under general anesthesia. The key elements of the study design, including patient selection, variable definition, and statistical modeling strategy, were predefined before data extraction and analysis. The study was reported with reference to the TRIPOD/TRIPOD+AI reporting recommendations for clinical prediction model studies, and a completed checklist is provided as Supplementary Table S1.

### Setting

The study was conducted at a tertiary teaching hospital located in Southwest China. Consecutive patients who underwent elective or non-emergency laparoscopic gynecologic surgery under general anesthesia between January 2019 and February 2024 were retrospectively identified from the hospital’s electronic medical record system and anesthesia information management system. The electronic records were queried using surgical procedure names and/or procedure codes related to laparoscopic gynecologic surgery, together with anesthesia records indicating general anesthesia. Retrieved records were cross-checked against operative notes and anesthesia records to confirm surgical approach, anesthesia type, temperature monitoring, and perioperative data availability. During this period, perioperative management protocols, including anesthetic techniques and temperature monitoring practices, remained largely stable. Data collection covered the entire perioperative period, from preoperative assessment to the immediate postoperative recovery phase.

### Participants

All consecutive adult patients (≥18 years) who underwent elective or non-emergency laparoscopic gynecologic surgery under general anesthesia during the study period were screened for eligibility. Accordingly, the model was derived from an elective or non-emergency gynecologic laparoscopic surgery cohort and was not intended to be generalized to emergency surgery, open surgery, non-gynecologic laparoscopic procedures, or broader general-anesthesia populations without further validation. Patients were included if they met all of the following criteria: underwent laparoscopic gynecologic surgery under general anesthesia; had available perioperative anesthesia records, including intraoperative or immediate postoperative temperature measurements; and had the clinical, demographic, and perioperative variables required for model development. Patients were excluded if they met any of the following criteria: underwent surgery without general anesthesia, such as regional anesthesia or monitored anesthesia care alone; underwent non-gynecologic or non-laparoscopic surgery; required planned postoperative admission to the intensive care unit with ongoing mechanical ventilation or therapeutic hypothermia; had documented severe preoperative neurological disorders affecting thermoregulation; or had missing key variables required for model development. A total of 512 potentially eligible records were initially identified from the electronic medical record and anesthesia information systems. After exclusion of 59 patients who did not meet the eligibility criteria and 29 patients with missing key variables, 424 patients were included in the final complete-case analysis. The 29 excluded records represented 6.4% of otherwise eligible patients after applying the clinical eligibility criteria and 5.7% of all initially screened records.

These patients were randomly divided into a training cohort (*n* = 294) for model development and a split-sample internal validation cohort (*n* = 130) for model assessment. No matching was performed. The patient-selection process is shown in Supplementary Figure S1.

### Outcome

The primary outcome was perioperative hypothermia, defined as any recorded core body temperature <36.0 °C occurring during the intraoperative period or within the immediate post-anesthesia care unit (PACU) recovery phase. Intraoperative temperature was monitored using clinically approved core temperature probes according to routine anesthetic practice. In this cohort, esophageal temperature monitoring was used in 286 patients (67.5%), nasopharyngeal temperature monitoring in 109 patients (25.7%), and bladder temperature monitoring in 29 patients (6.8%). Temperature values were automatically or manually documented in the anesthesia information management system at 15-min intervals during surgery, with additional measurements recorded when clinically indicated. For outcome classification, the presence of any recorded temperature value <36.0 °C was used to define perioperative hypothermia; the lowest recorded temperature was used for descriptive verification, whereas a time-weighted average temperature was not used for outcome definition. In the PACU, temperature was assessed using tympanic thermometry at admission and during early recovery, generally within the first 30 min after PACU arrival. Thus, perioperative hypothermia was classified if the patient had any intraoperative core temperature <36.0 °C or any PACU tympanic temperature <36.0 °C within this immediate recovery window.

### Predictors and covariates

Candidate variables were selected *a priori* based on clinical relevance and previous literature and included demographic variables, preoperative clinical variables, intraoperative variables, and perioperative management variables. Demographic variables included age (years) and body mass index (BMI, kg/m^2^). Preoperative clinical variables included ASA physical status classification, hemoglobin (g/L), and albumin (g/L). Intraoperative variables included operative time (minutes), estimated blood loss (mL), intraoperative fluid volume (mL), operating room temperature (°C), laparoscopic procedure type (simple vs. radical), and tumor type. The perioperative management variable was use of active warming (yes vs. no). All variables were defined before analysis, and no *post hoc* variable definitions were introduced. Because active warming is a treatment-related and decision-dependent intervention that may be initiated according to clinicians’ perceived risk of hypothermia, it was prespecified for exploratory and adjusted association analyses but was not considered eligible for inclusion in the final clinical nomogram. This approach was used to avoid incorporating a management decision variable into a prediction tool intended to support temperature-management decisions.

### Data sources and measurement

All data were extracted retrospectively from the hospital’s electronic medical record system and anesthesia information management system. Demographic and clinical data were recorded during routine preoperative assessment. Intraoperative variables, including operative time, fluid volume, blood loss, operating room temperature, active warming use, and serial temperature measurements, were documented prospectively by anesthesiology staff as part of standard clinical practice. Temperature monitoring site, recorded temperature values, and PACU temperature measurements were extracted from the anesthesia information management system and recovery-room nursing records. Several temperature-related or perioperative management variables were not consistently available in the retrospective electronic records, including preoperative baseline/core temperature, skin temperature, preoperative fasting duration, preoperative warming or prewarming status, detailed anesthetic drug exposure, and the temperature of insufflated carbon dioxide or irrigation fluids. These variables were therefore not included as candidate predictors and were considered as potential sources of residual confounding.

### Bias

Several strategies were used to minimize potential sources of bias. Consecutive patient inclusion was applied to reduce selection bias. Standardized institutional protocols for anesthesia management and temperature monitoring limited information bias. To address confounding, all clinically relevant variables were considered during multivariable modeling. Model development and validation were conducted using separate datasets to reduce the risk of overfitting.

### Study size

The study size was determined by the total number of eligible patients who underwent laparoscopic gynecologic surgery under general anesthesia during the predefined study period from January 2019 to February 2024 and met the inclusion criteria. Because this was a retrospective study using an existing clinical database, no prospective sample-size calculation was performed before data extraction. A total of 424 patients were included in the final complete-case analysis, of whom 279 developed perioperative hypothermia. Although the number of outcome events was considered in relation to the number of candidate and final predictors, sample-size adequacy was not justified by events per variable alone. In line with contemporary recommendations for developing clinical prediction models, including the pmsampsize framework proposed by Riley et al. (21), we additionally considered the number of final model parameters, the observed outcome proportion, and the need to limit model overfitting and optimism. The final model included three predictors—age, BMI, and operative time—resulting in a high number of outcome events relative to the final model complexity. In a *post hoc* assessment conceptually aligned with the pmsampsize framework, the available sample size was considered adequate for developing a parsimonious logistic prediction model with acceptable expected shrinkage and stable estimation. Nevertheless, because the cohort size was determined by available data rather than by a prospective sample-size calculation, this should be considered when interpreting the model and underscores the need for external validation.

### Assessment of linearity assumption

The linearity assumption for continuous predictors in the logistic regression model was assessed before final model construction. Restricted cubic spline functions were used to examine potential non-linear associations between age, BMI, operative time, and perioperative hypothermia on the logit scale. For each continuous predictor, spline-based models were compared with corresponding linear-term models, and evidence of non-linearity was evaluated using likelihood ratio tests. Visual inspection of spline plots was also performed. No strong evidence of clinically meaningful non-linearity was observed for age, BMI, or operative time; therefore, these variables were retained as linear continuous predictors in the final nomogram to preserve model parsimony and clinical interpretability.

### Quantitative variables

Continuous variables were analyzed as continuous measures without categorization whenever possible to preserve statistical information. Age, BMI, operative time, blood loss, fluid volume, hemoglobin, albumin, and operating room temperature were entered into regression models as continuous variables. No data-driven cut points were applied during model development.

### Statistical analysis

Baseline characteristics were summarized using medians with interquartile ranges (IQRs) for continuous variables and counts with percentages for categorical variables. Comparisons between the training and validation cohorts were performed using the Mann–Whitney U test for continuous variables and the chi-square test or Fisher’s exact test for categorical variables, as appropriate. Univariate logistic regression analyses were initially conducted to explore associations between candidate predictors and perioperative hypothermia. Subsequently, a multivariable logistic regression model including all candidate predictors was fitted to examine variables independently associated with perioperative hypothermia. Odds ratios (ORs) with 95% confidence intervals (CIs) were reported. To reduce dimensionality and enhance model robustness, feature selection was performed using least absolute shrinkage and selection operator (LASSO) regression and the Boruta algorithm. For LASSO regression, the cv.glmnet function from the glmnet package was used with family = “binomial” and ten-fold cross-validation in the training cohort. Continuous variables were standardized during model fitting using the default standardization procedure in glmnet. Categorical variables, including ASA physical status, tumor type, laparoscopic procedure type, and active warming, were converted into dummy variables before penalized regression, with clinically appropriate reference categories. Both λ_min and λ_1se were examined, and *λ*_min was selected for feature screening to avoid excluding potentially informative predictors in this modest-sized dataset. Variables with non-zero coefficients at λ_min were considered LASSO-selected candidates. LASSO coefficients at the selected λ are provided in Supplementary Table S3. The Boruta algorithm was performed using the Boruta package based on a random forest framework, with maxRuns = 500 and a fixed random seed (set.seed(202402)) to improve reproducibility. Variables confirmed as important by Boruta were compared with LASSO-selected variables, and predictors supported by both methods were reviewed for final model construction. Only variables that were consistently selected by the two methods and were conceptually appropriate for a risk-estimation tool were included in the final nomogram. Treatment-related or decision-dependent variables, particularly active warming, were not included in the final nomogram even if they showed statistical associations, because the model was intended to inform temperature-management decisions rather than incorporate such decisions as predictors. Before final model fitting, the linearity assumption for age, BMI, and operative time was assessed using restricted cubic spline terms in logistic regression models. Likelihood ratio tests and visual inspection of spline curves were used to evaluate potential departures from linearity. Because no strong evidence of clinically meaningful non-linearity was identified, the final model retained these predictors as linear continuous terms. A multivariable logistic regression model was developed in the training cohort and visualized using a nomogram. To support reproducibility, the final logistic prediction equation, including the model intercept and regression coefficients for age, BMI, and operative time, was reported in Supplementary Table S5. Model discrimination was assessed by calculating the area under the receiver operating characteristic curve (AUC). Calibration was evaluated using calibration plots, calibration-in-the-large intercept, calibration slope, integrated calibration index (ICI), expected-to-observed ratio (E/O ratio), Brier score, and the Hosmer–Lemeshow goodness-of-fit test. The calibration intercept was used to assess systematic underestimation or overestimation of predicted risk, whereas the calibration slope was used to assess potential overfitting or underfitting. The ICI summarized the average absolute difference between predicted and observed risks across the range of predicted probabilities. The Hosmer–Lemeshow test was reported as an additional numerical calibration assessment, with a non-significant result interpreted as no statistical evidence of poor fit.

Clinical utility was examined using decision curve analysis. The primary internal validation strategy used the prespecified split-sample validation cohort. In response to contemporary prediction-modeling recommendations and to reduce reliance on split-sample validation alone, an additional bootstrap internal validation analysis was performed using the full dataset. Bootstrap resampling with 500 replicates was used to estimate optimism in model discrimination and calibration. Optimism-corrected AUC, calibration slope, and Brier score were reported as supplementary internal validation metrics. Diagnostic performance was summarized using sensitivity, specificity, accuracy, positive predictive value, and negative predictive value. The classification threshold was determined in the training cohort using the Youden index and then applied unchanged to the validation cohort to assess transportability. Although a validation-cohort Youden cutoff was explored descriptively, the primary validation classification metrics were calculated using the training-derived cutoff. A complete-case analysis was performed. Patients with missing key variables required for model development or outcome classification (*n* = 29) were excluded before cohort allocation; therefore, no imputation of missing data was performed. The missing-data proportion was 6.4% among otherwise eligible patients after clinical exclusions and 5.7% among all initially screened records. Complete-case analysis was chosen because the proportion of missing key variables was relatively low and because these variables were essential for model development, validation, or outcome definition. Nevertheless, the possibility of selection bias due to complete-case analysis could not be fully excluded and is acknowledged as a limitation. The number of patients excluded because of missing key variables is shown in the participant flow diagram. No subgroup analyses or additional sensitivity analyses were conducted. Reporting of the modeling workflow followed TRIPOD/TRIPOD+AI-related elements, including specification of the data source, eligibility criteria, outcome definition, candidate predictors, feature-selection procedures, model performance measures, internal validation approach, and missing-data handling. All statistical analyses were performed using R software (version 4.2.2; R Foundation for Statistical Computing, Vienna, Austria). Key R packages included glmnet for LASSO regression, Boruta for feature selection, rms for nomogram construction and calibration, pROC for receiver operating characteristic analysis, and rmda for decision curve analysis. A two-sided *p* value < 0.05 was considered statistically significant.

## Results

### Baseline characteristics of the training and validation cohorts

The baseline characteristics of patients in the training cohort (*n* = 294) and the validation cohort (*n* = 130) are summarized descriptively in Table 1. These comparisons were intended to describe the distribution and balance of patient characteristics after random allocation, rather than to serve as formal hypothesis testing. Overall, the training and validation cohorts showed broadly similar distributions of demographic, perioperative, and clinical variables. Median age, body mass index, operative time, intraoperative blood loss, operating room temperature, intraoperative fluid volume, hemoglobin level, and albumin concentration were comparable between the training and validation cohorts (all *p* > 0.05). In addition, the distributions of ASA class, use of active warming, tumor type, and laparoscopic procedure type did not differ significantly between the two cohorts. Importantly, the proportion of patients who developed perioperative hypothermia was similar in the training cohort (67.7%) and the validation cohort (61.5%) (*p* = 0.263). The overall incidence of perioperative hypothermia was 65.8%, which should be interpreted in light of the outcome definition based on any core temperature <36.0 °C during the intraoperative or immediate postoperative period. The high observed incidence may also reflect the intensive temperature surveillance used in this cohort, with intraoperative temperature values recorded at 15-min intervals and additional PACU temperature assessment during early recovery. In addition, the median operating-room temperature was 22.60 °C in both cohorts, the median operative time exceeded 160 min, and active warming was not used in approximately one-third of patients. These features may have increased the likelihood of detecting hypothermic episodes and should be considered when comparing the incidence with that reported in other surgical populations. These descriptive findings suggest that the random allocation strategy yielded two broadly comparable cohorts for model development and split-sample internal validation.

**Table 1:** Comparison of baseline characteristics between the training and validation cohorts.

Variable	Training cohort (*n* = 294)	Validation cohort (*n* = 130)	*p* value
Age (years)	55.00 (43.00–66.00)	57.00 (44.25–66.75)	0.433
Body mass index (kg/m^2^)	23.50 (21.42–26.20)	24.20 (21.52–26.90)	0.181
Operative time (min)	178.50 (122.25–237.00)	167.50 (108.25–228.75)	0.294
Blood loss (mL)	446.00 (269.25–627.00)	469.00 (216.00–612.50)	0.753
Operating room temperature (°C)	22.60 (21.60–23.60)	22.60 (21.63–23.37)	0.620
Fluid volume (mL)	2337.00 (1490.50–3066.25)	2072.00 (1340.00–3033.75)	0.225
Hemoglobin (g/L)	120.50 (109.38–129.60)	119.50 (112.03–129.97)	0.900
Albumin (g/L)	39.75 (37.20–42.10)	40.00 (37.23–42.88)	0.579
ASA class			0.184
ASA I	74 (25.2%)	42 (32.3%)	
ASA II	132 (44.9%)	47 (36.2%)	
ASA III	88 (29.9%)	41 (31.5%)	
Active warming used			0.574
No	92 (31.3%)	45 (34.6%)	
Yes	202 (68.7%)	85 (65.4%)	
Tumor type			0.967
Cervical	102 (34.7%)	46 (35.4%)	
Endometrial	85 (28.9%)	36 (27.7%)	
Ovarian	107 (36.4%)	48 (36.9%)	
Laparoscopic type			0.174
Radical	131 (44.6%)	48 (36.9%)	
Simple	163 (55.4%)	82 (63.1%)	
Perioperative hypothermia			0.263
No	95 (32.3%)	50 (38.5%)	
Yes	199 (67.7%)	80 (61.5%)	

Continuous variables are presented as median (interquartile range, IQR) and were compared using the Mann–Whitney U test. Categorical variables are presented as number (percentage) and were compared using the chi-square test or Fisher’s exact test, as appropriate. Perioperative hypothermia was defined as any recorded temperature <36.0 °C during the intraoperative period or within the immediate PACU recovery phase. A two-sided *p* value < 0.05 was considered statistically significant.

### Exploratory univariate and multivariable logistic regression analyses

Exploratory univariate and multivariable logistic regression analyses examining associations between candidate variables and perioperative hypothermia are presented in Table 2. These analyses were conducted to characterize candidate-variable associations and to inform clinical interpretation; they were not intended to define the final prediction equation. In univariate analyses, increasing age (OR = 1.05 per year, 95% CI 1.04–1.07, *p* < 0.001), higher ASA class (ASA III vs. I: OR = 2.12, 95% CI 1.24–3.64, *p* = 0.006), longer operative time (OR = 1.01 per minute, 95% CI 1.00–1.01, *p* < 0.001), lower body mass index (OR = 0.86, 95% CI 0.81–0.91, *p* < 0.001), and absence of active warming (OR = 0.50, 95% CI 0.31–0.78, *p* = 0.003) were significantly associated with an increased risk of perioperative hypothermia. Other variables, including albumin level, hemoglobin concentration, blood loss, operating room temperature, fluid volume, laparoscopic type, and tumor type, did not show statistically significant associations in univariate analyses. In the multivariable logistic regression model adjusting for all listed covariates simultaneously, age remained independently associated with perioperative hypothermia (adjusted OR = 1.07 per year, 95% CI 1.05–1.09, *p* < 0.001). Patients with ASA class III had higher odds of perioperative hypothermia than those classified as ASA I (adjusted OR = 2.63, 95% CI 1.37–5.11, *p* = 0.004). Lower body mass index and longer operative time were also independently associated with perioperative hypothermia (BMI: adjusted OR = 0.82 per kg/m^2^, 95% CI 0.76–0.88, *p* < 0.001; operative time: adjusted OR = 1.01 per minute, 95% CI 1.01–1.01, *p* < 0.001). In contrast, the use of active warming was independently associated with lower odds of perioperative hypothermia (adjusted OR = 0.39, 95% CI 0.22–0.67, *p* < 0.001). Fluid volume showed a statistically significant but modest association in the multivariable model (adjusted *p* = 0.027), whereas albumin, hemoglobin, blood loss, operating room temperature, laparoscopic type, and tumor type were not independently associated with perioperative hypothermia after adjustment. Accordingly, variables that were statistically significant in this exploratory multivariable association model, such as ASA class, fluid volume, and active warming, were not automatically retained in the final nomogram. The final clinical prediction model was specified separately after LASSO/Boruta-based feature selection and prespecified conceptual review, and included only age, BMI, and operative time.

**Table 2:** Univariate and multivariable logistic regression analyses for perioperative hypothermia.

Variable	Univ OR (95% CI)	Univ *p*	Multi OR (95% CI)	Multi *p*
Age (years)	1.05 (1.04–1.07)	<0.001	1.07 (1.05–1.09)	<0.001
Albumin (g/L)	0.98 (0.93–1.04)	0.485	0.99 (0.92–1.06)	0.705
ASA class				
ASA II vs. I	1.47 (0.91–2.37)	0.119	1.47 (0.82–2.66)	0.195
ASA III vs. I	2.12 (1.24–3.64)	0.006	2.63 (1.37–5.11)	0.004
Blood loss (mL)	1.00 (1.00–1.00)	0.991	1.00 (1.00–1.00)	0.547
Body mass index (kg/m^2^)	0.86 (0.81–0.91)	<0.001	0.82 (0.76–0.88)	<0.001
Fluid volume (mL)	1.00 (1.00–1.00)	0.335	1.00 (1.00–1.00)	0.027
Hemoglobin (g/L)	1.00 (0.98–1.01)	0.605	0.99 (0.98–1.01)	0.239
Laparoscopic type (simple vs. radical)	0.91 (0.60–1.36)	0.646	0.81 (0.50–1.33)	0.414
Operative time (min)	1.01 (1.00–1.01)	<0.001	1.01 (1.01–1.01)	<0.001
Operating room temperature (°C)	1.03 (0.89–1.18)	0.697	1.00 (0.85–1.19)	0.977
Tumor type				
Endometrial vs. cervical	0.90 (0.54–1.49)	0.676	1.00 (0.54–1.85)	0.994
Ovarian vs. cervical	0.95 (0.59–1.53)	0.842	1.10 (0.62–1.96)	0.746
Active warming used (yes vs. no)	0.50 (0.31–0.78)	0.003	0.39 (0.22–0.67)	<0.001

Values are presented as odds ratios (ORs) with 95% confidence intervals (CIs). Univariate and multivariable analyses were performed using logistic regression models.

Perioperative hypothermia was defined as any recorded temperature <36.0 °C during the intraoperative period or within the immediate PACU recovery phase. In multivariable analysis, all listed variables were entered simultaneously into the model. For categorical variables, the reference categories were ASA I, cervical cancer, radical laparoscopic surgery, and no active warming. A two-sided *p* value < 0.05 was considered statistically significant.

### Assessment of linearity for continuous predictors

Restricted cubic spline analyses were performed to assess whether the associations of age, BMI, and operative time with perioperative hypothermia deviated from linearity on the logit scale. Visual inspection of the spline curves did not suggest marked non-linear patterns. Likelihood ratio tests did not provide strong evidence of non-linearity for age (*p* for non-linearity = 0.214), BMI (*p* for non-linearity = 0.168), or operative time (*p* for non-linearity = 0.091). Therefore, these predictors were modeled as linear continuous variables in the final logistic regression model and nomogram.

### Feature selection and model development

**Figure 1:**
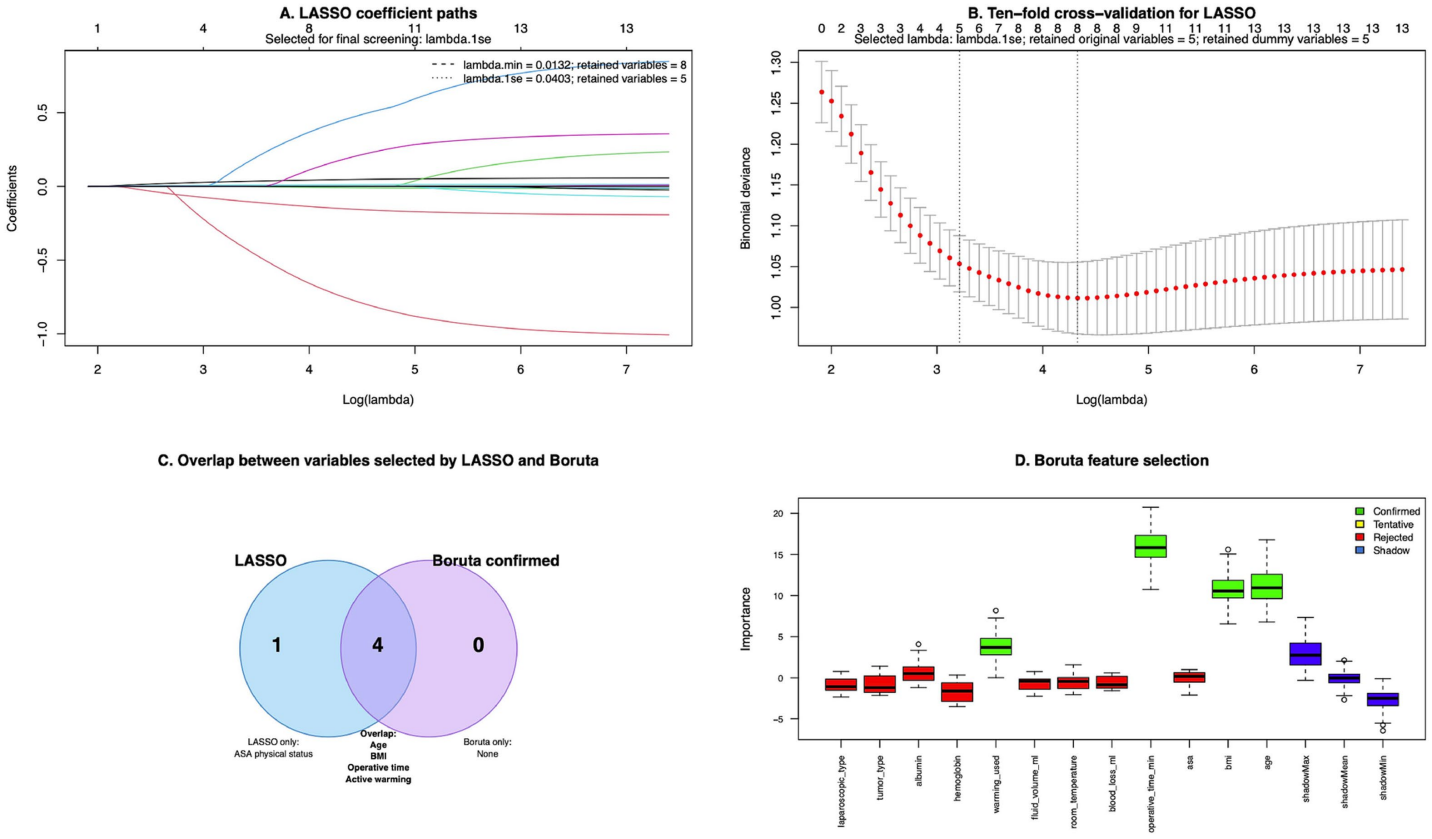
Feature selection using LASSO and Boruta algorithms. **(A)** LASSO regression coefficient paths of the candidate predictors. Each colored line represents the coefficient trajectory of an individual variable as a function of the log-transformed regularization parameter (log *λ*). The vertical dashed lines indicate λ_min_ and λ_1se_, and the selected λ value and number of retained variables are annotated on the panel. Candidate variables for subsequent screening were retained based on non-zero coefficients at λ_min_. **(B)** Ten-fold cross-validation results of the LASSO regression model. The red dots represent the mean binomial deviance at each λ value, and the error bars denote ±1 standard error. The two vertical dashed lines correspond to λ_min_ and λ_1se_, respectively. The number of non-zero coefficients retained at each λ is shown along the upper axis, and the selected λ value is annotated on the panel. **(C)** Venn diagram illustrating the overlap between variables selected by LASSO regression and those confirmed by the Boruta algorithm. The overlapping variables were age, body mass index, and operative time. LASSO-only variables and Boruta-only variables, if any, are explicitly displayed in the diagram. **(D)** Boruta feature selection results displayed as boxplots of variable importance. Green boxes indicate confirmed variables, yellow boxes indicate tentative variables, red boxes indicate rejected variables, and blue boxes represent shadow features. The importance of each original variable is compared against randomized shadow variables to assess statistical relevance.

To identify the most informative predictors for model construction and to reduce potential multicollinearity, feature selection was performed using both the least absolute shrinkage and selection operator (LASSO) regression and the Boruta algorithm. As shown in Figures 1A,B, LASSO regression using cv.glmnet with family = “binomial” and ten-fold cross-validation was applied to the training cohort. Both λ_min and λ_1se were displayed in the LASSO plot, and λ_min was used for candidate predictor screening. At λ_min, predictors with non-zero coefficients were retained as LASSO-selected variables; the corresponding coefficients are presented in Supplementary Table S3. In parallel, the Boruta algorithm was used to assess variable importance based on a random forest framework (Figure 1D). Variables consistently identified as important by Boruta were considered robust predictors. The overlap between predictors selected by LASSO and those confirmed by Boruta is illustrated in Figure 1C. Based on the concordant feature-selection results and prespecified conceptual considerations for a clinical risk-estimation tool, age, body mass index, and operative time were selected as the final predictors for subsequent model development. Although ASA physical status and active warming were statistically associated with perioperative hypothermia in the exploratory multivariable association model shown in Table 2, they were not included in the final nomogram. ASA physical status was not retained because its incremental contribution to the parsimonious prediction model was limited after accounting for age, BMI, and operative time, and because the number of ASA III patients was relatively limited. Active warming was not included because it is a treatment-related and decision-dependent management variable that may be initiated according to clinicians’ perceived hypothermia risk; incorporating it into a model intended to guide temperature-management decisions could reduce interpretability and introduce circularity. The final nomogram was therefore constructed as a separate three-predictor logistic regression model rather than as the full multivariable association model shown in Table 2.

### Development of the nomogram

**Figure 2:**
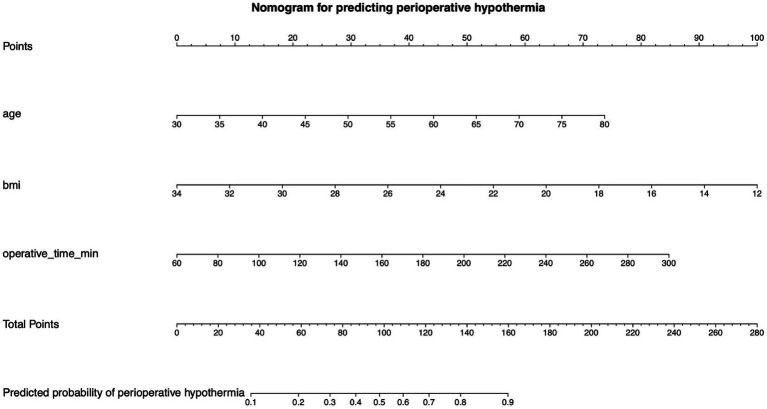
Nomogram for predicting perioperative hypothermia. A nomogram was constructed based on the final multivariable logistic regression model incorporating age, body mass index (BMI), and operative time. Each predictor is assigned a corresponding point value according to its contribution to the model. The total points derived from summing individual predictor scores correspond to a predicted probability of perioperative hypothermia. The nomogram provides an individualized risk estimation for clinical use.

A multivariable logistic regression model incorporating only the three selected predictors—age, body mass index, and operative time—was constructed using the training cohort as the final prediction model. To support reproducibility, external validation, and potential model updating, the full parameters of the final three-predictor logistic regression model, including the intercept, regression coefficients, standard errors, odds ratios, and 95% confidence intervals, are provided in Supplementary Table S5. The linear predictor was calculated as LP = −0.214 + 0.059 × age − 0.176 × BMI + 0.010 × operative time, and the predicted probability of perioperative hypothermia was calculated as *p* = 1 / [1 + exp.(−LP)]. Because the model has not yet undergone external validation, we did not develop an online calculator at this stage; however, the explicit equation and full model coefficients allow independent reproduction, external validation, recalibration, and future updating. Based on this model, a nomogram was developed to provide an individualized and user-friendly tool for estimating the probability of perioperative hypothermia (Figure 2). Each predictor was assigned a weighted point value proportional to its regression coefficient, and the total score corresponded to an estimated risk of perioperative hypothermia. The nomogram enables straightforward bedside risk estimation by summing the points for each predictor and projecting the total score to the predicted probability scale.

### Model discrimination, calibration, and clinical utility

**Figure 3:**
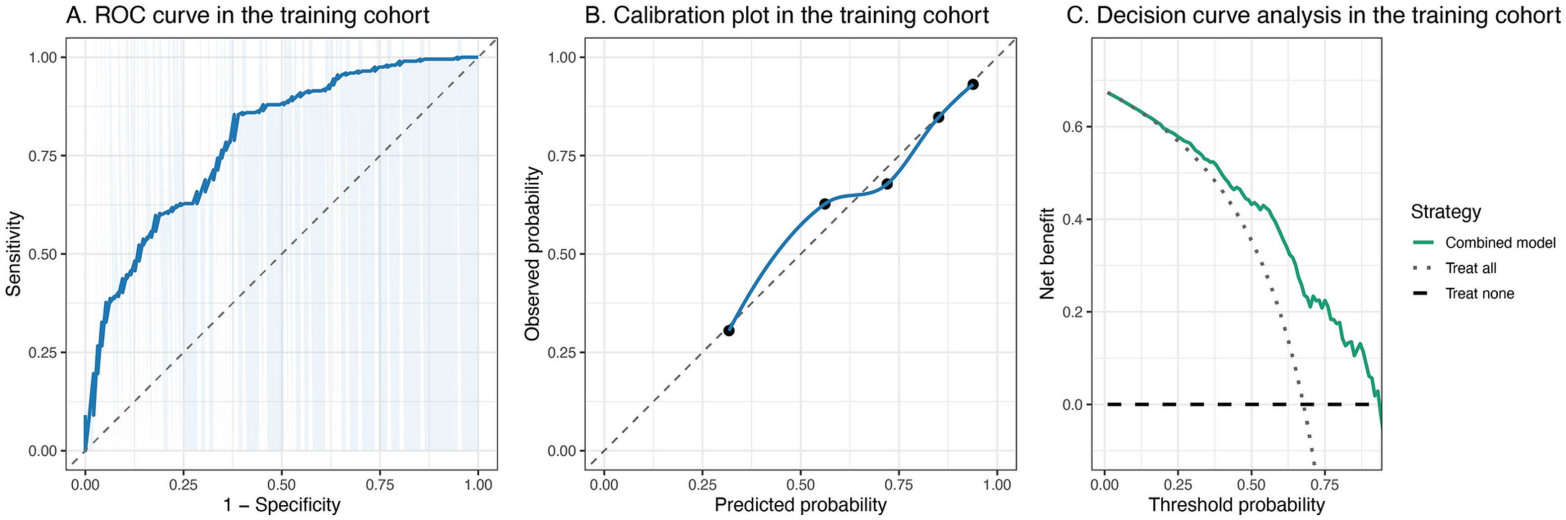
Model performance in the training cohort. **(A)** Receiver operating characteristic (ROC) curve of the prediction model in the training cohort. The shaded area represents the area under the ROC curve (AUC), with the corresponding 95% confidence interval (CI) displayed. **(B)** Calibration plot of the model in the training cohort. The apparent curve reflects model-predicted probabilities, the bias-corrected curve is obtained via bootstrap resampling, and the dashed diagonal line represents perfect calibration. Numerical calibration metrics were: calibration intercept = 0.01, calibration slope = 0.98, ICI = 0.035, E/O ratio = 1.00, Brier score = 0.192, and Hosmer–Lemeshow χ^2^ = 6.84, *p* = 0.554. **(C)** Decision curve analysis (DCA) in the training cohort. Net benefit is plotted against a range of threshold probabilities for individual predictors (age, BMI, operative time) and for the combined model (“All”). The horizontal dashed line indicates the strategy of treating no patients, and the sloped line represents treating all patients.

**Figure 4:**
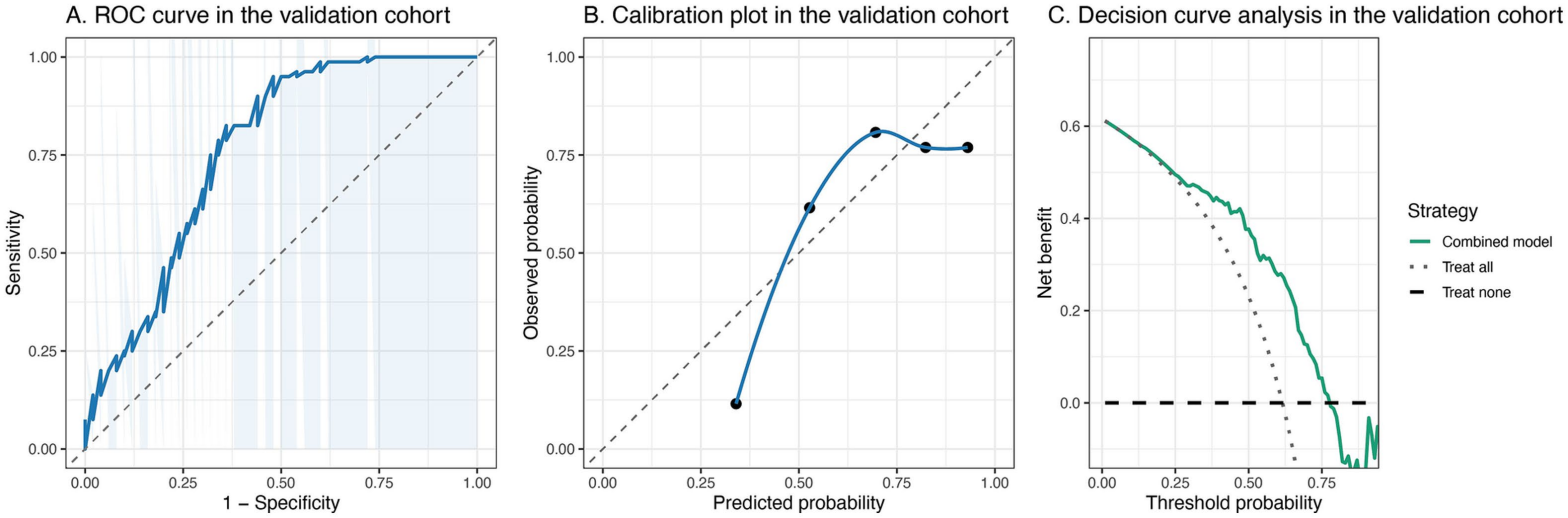
Model performance in the validation cohort. **(A)** Receiver operating characteristic (ROC) curve of the prediction model in the independent validation cohort, with AUC and 95% CI reported. **(B)** Calibration plot in the validation cohort, showing agreement between predicted and observed probabilities of perioperative hypothermia. Numerical calibration metrics were: calibration intercept = −0.04, calibration slope = 0.93, ICI = 0.046, E/O ratio = 0.98, Brier score = 0.187, and Hosmer–Lemeshow χ^2^ = 7.31, *p* = 0.503. **(C)** Decision curve analysis (DCA) in the validation cohort. The net clinical benefit of the combined model across a range of threshold probabilities is compared with individual predictors and default strategies (treat-all and treat-none), indicating the potential clinical utility of the model.

The discriminative performance, calibration, and clinical utility of the prediction model were evaluated in both the training and validation cohorts. In the training cohort (Figure 3A), the model demonstrated good discrimination, with an area under the receiver operating characteristic curve (AUC) of 0.788 (95% CI 0.732–0.843). Calibration analysis showed good agreement between predicted and observed probabilities, as indicated by the proximity of the apparent and bias-corrected calibration curves to the ideal reference line (Figure 3B). Numerical calibration metrics further supported acceptable calibration. In the training cohort, the calibration intercept was 0.01, the calibration slope was 0.98, the ICI was 0.035, the E/O ratio was 1.00, the Brier score was 0.192, and the Hosmer–Lemeshow test showed no evidence of poor fit (χ^2^ = 6.84, *p* = 0.554). In the validation cohort, the calibration intercept was −0.04, the calibration slope was 0.93, the ICI was 0.046, the E/O ratio was 0.98, the Brier score was 0.187, and the Hosmer–Lemeshow test also suggested acceptable fit (χ^2^ = 7.31, *p* = 0.503). These findings suggested no major systematic miscalibration, although the validation calibration slope below 1.0 indicated mild overfitting, consistent with the bootstrap optimism-corrected calibration slope of 0.91. Detailed numerical calibration metrics are shown in Supplementary Table S4. Decision curve analysis suggested that the model provided a net clinical benefit across a range of clinically relevant risk thresholds (Figure 3C). In the independent validation cohort, model performance remained stable. The AUC was 0.810 (95% CI 0.727–0.893), indicating preserved discriminative ability (Figure 4A). Calibration curves again demonstrated good concordance between predicted and observed risks (Figure 4B), and decision curve analysis confirmed favorable clinical utility in the validation dataset (Figure 4C).

The diagnostic performance of the model at the training-derived cutoff is summarized in Table 3. The optimal cutoff was determined in the training cohort using the Youden index and then applied unchanged to the validation cohort. The training-derived cutoff was −0.2187 on the logit scale, corresponding to a predicted probability threshold of 0.446. Therefore, within this study setting, patients with an estimated risk of approximately ≥45% may be considered as a higher-risk group for intensified temperature monitoring and consideration of active warming. In the training set, this cutoff yielded a sensitivity of 0.623, specificity of 0.853, accuracy of 0.697, PPV of 0.899, and NPV of 0.519. When the same cutoff was applied to the validation cohort, the model achieved a sensitivity of 0.925, specificity of 0.580, accuracy of 0.792, PPV of 0.779, and NPV of 0.829. Because the model has not yet undergone external validation, this threshold should be interpreted as a pragmatic risk-stratification threshold rather than as a definitive treatment threshold. These findings provide a more conservative assessment of classification performance using a fixed training-derived threshold. The difference in sensitivity and specificity between the training and validation cohorts should be interpreted as threshold-dependent classification behavior, rather than as evidence of superior performance in the validation cohort. Collectively, these findings indicate that the model demonstrates robust discrimination, good calibration, and consistent clinical usefulness when applied to an independent validation cohort.

**Table 3:** Diagnostic performance of the model at the training-derived Youden cutoff.

Dataset	Cutoff on logit scale	Probability threshold	Sensitivity	Specificity	Accuracy	PPV	NPV	Youden index
Training set	−0.2187	0.446	0.623	0.853	0.697	0.899	0.519	0.476
Validation set	−0.2187	0.446	0.925	0.580	0.792	0.779	0.829	0.505

The cutoff was determined in the training cohort using the Youden index and then applied unchanged to the validation cohort. The cutoff of −0.2187 on the logit scale corresponds to a predicted probability threshold of 0.446 using *p* = 1 / [1 + exp(−LP)]. Diagnostic performance was calculated by defining predicted perioperative hypothermia as the positive classification. In the training cohort, the cutoff classified 124 true-positive, 75 false-negative, 81 true-negative, and 14 false-positive cases. In the validation cohort, the cutoff classified 74 true-positive, 6 false-negative, 29 true-negative, and 21 false-positive cases. This threshold may be used as a pragmatic risk-stratification threshold within the study setting, rather than as a mandatory treatment threshold. PPV, positive predictive value; NPV, negative predictive value; LP, linear predictor.

### Bootstrap optimism-corrected internal validation

To complement the split-sample validation analysis, bootstrap internal validation was performed using the full cohort. In the full dataset, the apparent AUC of the final three-predictor model was 0.794. After 500 bootstrap resamples, the estimated optimism in AUC was 0.018, resulting in an optimism-corrected AUC of 0.776. The optimism-corrected calibration slope was 0.91, suggesting mild overfitting but acceptable overall model stability. The optimism-corrected Brier score was 0.197. These bootstrap results were broadly consistent with the split-sample validation findings and supported the internal robustness of the parsimonious model.

## Discussion

In this single-center retrospective cohort study, we developed and internally validated a parsimonious prediction model for perioperative hypothermia in adult patients undergoing laparoscopic gynecologic surgery under general anesthesia. By applying complementary feature selection methods and internal validation, we retained age, body mass index (BMI), and operative time as the final model predictors and integrated them into a user-friendly nomogram. The model demonstrated good discrimination, satisfactory calibration, and consistent clinical utility in both the training and validation cohorts, supporting its potential value for individualized perioperative risk stratification within this surgical setting. Because this study was retrospective and observational, all predictor–outcome relationships should be interpreted as associations useful for risk estimation rather than as causal effects.

The overall incidence of perioperative hypothermia in this cohort was 65.8%, which is relatively high compared with many perioperative reports but remains compatible with the upper range reported in some laparoscopic and gynecologic surgical populations. Several factors may explain this finding. First, the outcome was defined as any recorded core temperature <36.0 °C during the intraoperative period or any immediate PACU temperature <36.0 °C, which may capture transient or short-duration hypothermic episodes that might be missed in studies using fewer measurement points or only postoperative assessments. Second, temperature surveillance was relatively intensive, with intraoperative temperature values recorded at 15-min intervals and PACU temperature assessment during early recovery, thereby increasing the likelihood of detecting hypothermia. Third, the median operating-room temperature was approximately 22.6 °C, and active warming was not used in about one-third of patients, which may have contributed to heat loss in susceptible patients. Fourth, the cohort had a relatively long operative-time profile, with median operative times exceeding 160 min in both training and validation cohorts. Prolonged laparoscopic gynecologic surgery may involve sustained anesthetic-induced thermoregulatory impairment, Trendelenburg positioning, pneumoperitoneum, pelvic exposure, and variable irrigation or fluid administration. Therefore, the observed incidence should be interpreted in relation to the outcome definition, surveillance intensity, operating-room environment, warming practices, and procedure-specific characteristics of this cohort, rather than as a direct estimate for all laparoscopic or gynecologic surgical populations.

Age was retained as one of the final predictors in the nomogram, with older age corresponding to a higher predicted probability of perioperative hypothermia in this cohort. This finding should be interpreted as a predictive association rather than evidence that age itself causally determines hypothermia risk. The observed association is consistent with physiological mechanisms described in previous literature, which suggest that older patients may have reduced thermoregulatory reserve, attenuated vasoconstrictive responses, and lower metabolic heat production during anesthesia (22, 23). These mechanisms may help explain why age contributed to risk stratification in the present model, but they were not directly tested in this retrospective study.

BMI was also retained as a final model predictor, with lower BMI associated with a higher predicted probability of perioperative hypothermia. This association is clinically plausible and is consistent with prior studies suggesting that patients with lower body mass or reduced body composition reserves may have less subcutaneous insulation and lower thermal reserve during anesthesia (24, 25). However, the present study did not directly measure body composition, heat transfer, or metabolic heat production. Therefore, BMI should be interpreted as a practical predictor for risk estimation rather than as evidence of a causal pathway linking lower BMI to perioperative hypothermia (26).

Operative time was the third predictor retained in the final model, with longer procedures associated with a higher predicted probability of perioperative hypothermia. This predictive association is consistent with previous reports linking prolonged surgery to greater cumulative exposure to anesthetic-induced thermoregulatory impairment, operating-room environmental conditions, and procedural heat loss (27, 28). In laparoscopic gynecologic surgery, longer operative time may also be a marker of greater procedural complexity, prolonged pneumoperitoneum, Trendelenburg positioning, pelvic exposure, and variable irrigation or fluid administration. These factors provide plausible clinical explanations for the observed association, but the retrospective design of this study does not allow causal inference regarding operative duration or specific intraoperative mechanisms (29).

Several variables, including ASA physical status, fluid volume, blood loss, and active warming, showed associations with perioperative hypothermia in univariate or multivariable analyses but were not included in the final nomogram. This distinction reflects the different purposes of an explanatory association model and a parsimonious clinical prediction model. The exploratory multivariable model in Table 2 was used to characterize adjusted associations among candidate variables, whereas the final nomogram was developed after feature selection, assessment of incremental predictive value, and prespecified clinical review. This exclusion should not be interpreted as evidence that these factors are clinically unimportant. Rather, the final model was designed as a parsimonious risk-estimation tool using variables that were both statistically supported and conceptually appropriate for prediction. Active warming deserves particular consideration. Although active warming was associated with lower odds of perioperative hypothermia in the adjusted regression analysis, this association should not be interpreted as a causal effect in the present retrospective study. In routine practice, active warming may be preferentially applied to patients whom anesthesiologists already perceive to be at higher baseline risk, creating the possibility of confounding by indication. Conversely, effective warming may reduce the observed occurrence of hypothermia among treated patients. Because active warming is a clinical management decision and may lie on the pathway between perceived risk and observed temperature outcome, including it in the final nomogram could reduce interpretability and limit the model’s usefulness for guiding temperature-management decisions. Therefore, active warming was evaluated in association analyses but was prespecified as ineligible for inclusion in the final clinical nomogram.

Compared with existing models, the main practical feature of the present nomogram is its parsimony. The final model uses only three variables that are routinely available before or during surgery and do not require specialized laboratory testing, complex scoring systems, or postoperative information. This design may facilitate bedside risk estimation and integration into routine anesthetic workflow. Nevertheless, because different models were developed in different surgical populations and with different predictor sets, direct superiority over existing tools cannot be inferred from the present study. External validation and head-to-head comparison with established risk scores or previously published models are needed before broader implementation.

We acknowledge that the present study did not perform a formal head-to-head comparison with established perioperative hypothermia risk scores, such as the Maurtua score, or with other previously published hypothermia prediction models. This limits our ability to claim incremental predictive value over existing tools. Several reasons should be considered when interpreting this omission. First, some variables required by established scores were not consistently available in our retrospective electronic records, which would have resulted in additional missingness and potential selection bias. Second, existing tools were developed in broader surgical populations or different perioperative contexts, whereas the present model was designed specifically for adult patients undergoing laparoscopic gynecologic surgery under general anesthesia. Third, our primary aim was to develop an internally validated, parsimonious, setting-specific nomogram using routinely available variables rather than to establish superiority over existing risk scores. Future multicenter external validation studies should directly compare this nomogram with established hypothermia risk tools and assess whether it provides improved calibration, discrimination, clinical net benefit, or implementation feasibility in laparoscopic gynecologic surgery.

The present study should be interpreted in the context of an expanding literature on prediction models and nomograms for perioperative or intraoperative hypothermia. Recent studies have developed prediction tools in several surgical settings, including robotic surgery, gynecological surgery, video-assisted thoracoscopic surgery, general laparoscopic surgery, and gynecological laparoscopic surgery populations (30, 31). In addition, a recent systematic review has summarized the methodological characteristics and limitations of available perioperative hypothermia prediction models (32). Therefore, the present study does not claim to be the first prediction model in this field. Rather, it provides a complementary, setting-specific nomogram for adult patients undergoing laparoscopic gynecologic surgery under general anesthesia. Its main added value lies in the construction of a parsimonious model using only three routinely available predictors—age, BMI, and operative time—together with complementary LASSO and Boruta feature-selection procedures and evaluation of discrimination, calibration, and decision curve analysis in a split-sample internal validation cohort. This design may facilitate clinical interpretability and bedside implementation, although external validation and comparison with existing models are still required before broader use.

The nomogram developed in this study provides a simple and intuitive tool for estimating the individual probability of perioperative hypothermia among adult patients undergoing laparoscopic gynecologic surgery under general anesthesia, which is the population in which the model was derived and internally validated. In this specific surgical setting, the training-derived Youden threshold corresponded to an estimated probability of approximately 45%. Patients with a predicted risk at or above this level may be considered for intensified temperature surveillance, earlier initiation of active warming, warmed intravenous fluids when appropriate, and closer PACU temperature follow-up. This threshold is intended to support pragmatic risk stratification and quality-improvement audits rather than to mandate a fixed intervention. Its clinical impact requires prospective evaluation before routine implementation. The model is intended to support risk stratification and clinical decision-making rather than to replace clinical judgment or prescribe mandatory interventions. Because the cohort was restricted to laparoscopic gynecologic surgery, the model should not be directly extrapolated to non-gynecologic laparoscopic procedures or broader general-anesthesia populations without external validation.

This study has several strengths. First, it included a relatively large cohort of patients undergoing laparoscopic surgery under general anesthesia, with standardized perioperative management practices over the study period. Second, the use of complementary feature selection methods and an independent validation cohort enhanced the robustness and reliability of the model. Third, adherence to contemporary methodological standards for prediction modeling strengthens the transparency and reproducibility of the findings.

Several limitations should also be acknowledged. First, the retrospective and single-center design limits causal inference and may restrict generalizability to other institutions, geographic regions, and surgical populations outside elective or non-emergency laparoscopic gynecologic surgery. In particular, the model should not be assumed to apply to emergency surgery, open surgery, non-gynecologic laparoscopic surgical populations such as gastrointestinal, urological, or thoracic surgery patients, or broader general-anesthesia populations without external validation. Second, the sample size was determined by the number of eligible patients available during the predefined study period rather than by a prospective sample-size calculation based on contemporary prediction-modeling criteria. Although a *post hoc* assessment considering model complexity, outcome proportion, and expected overfitting suggested that the available sample was adequate for this parsimonious model, the absence of a prespecified sample-size calculation remains a methodological limitation. Third, the original internal validation strategy used a split-sample design, which can produce unstable performance estimates in modest-sized datasets and may use the available data less efficiently than resampling-based validation. To address this limitation, we supplemented the split-sample analysis with bootstrap optimism-corrected internal validation using the full cohort, and the results were broadly consistent with the split-sample findings. Nevertheless, the nomogram has only been internally validated and has not yet undergone external validation in an independent cohort or formal comparison with established perioperative hypothermia risk scores. Therefore, it should be regarded as a preliminary risk-estimation tool and should not be deployed for routine clinical decision-making without further external validation, recalibration if necessary, and prospective assessment of clinical impact. Fourth, residual confounding cannot be excluded. Some potentially relevant variables were not consistently available in the retrospective database, including preoperative baseline/core temperature, skin temperature, preoperative fasting duration, preoperative warming or prewarming status, detailed anesthetic drug exposure, intraoperative ambient humidity, and the temperature of insufflated carbon dioxide or irrigation fluids. These unmeasured factors may have influenced both hypothermia occurrence and clinicians’ decisions regarding temperature management. Fifth, a complete-case analysis was used after excluding 29 patients with missing key variables, corresponding to 6.4% of otherwise eligible records and 5.7% of all initially screened records. Although the missing proportion was relatively low and complete-case analysis was considered acceptable for this retrospective model-development study, selection bias related to missing data cannot be fully excluded. Sixth, information bias may have occurred because temperature-monitoring sites varied across patients, including esophageal, nasopharyngeal, bladder, and PACU tympanic measurements. Although these methods were used as part of routine anesthetic practice and temperature values were generally recorded at 15-min intervals during surgery, differences in monitoring site, measurement frequency, or additional clinically indicated measurements may have affected outcome classification. Finally, although ASA physical status was included in the exploratory association analyses, the number of ASA III patients was limited. This may reduce the precision of risk estimates in higher-risk patients and limit extrapolation to patients with more severe systemic disease. Future studies with larger, multicenter cohorts, broader ASA distributions, standardized temperature-monitoring protocols, and external validation are needed to confirm model transportability and clinical usefulness.

In conclusion, we developed and internally validated a concise nomogram incorporating age, BMI, and operative time to estimate the risk of perioperative hypothermia specifically in adult patients undergoing laparoscopic gynecologic surgery under general anesthesia. Within this surgical setting, the model demonstrated stable internal performance and potential clinical utility for individualized perioperative temperature-risk assessment. Future studies should focus on external validation in independent laparoscopic gynecologic cohorts, comparison with established perioperative hypothermia risk tools, and prospective evaluation of clinical impact before broader implementation.

## Data Availability

The original contributions presented in the study are included in the article/Supplementary material, further inquiries can be directed to the corresponding authors.

## References

[1] Sessler DI. Perioperative thermoregulation and heat balance. Lancet. (2016) 387:2655–64. doi: 10.1016/S0140-6736(15)00981-2, 26775126

[2] Rauch S Miller C Bräuer A Wallner B Bock M Paal P. Perioperative hypothermia-a narrative review. Int J Environ Res Public Health. (2021) 18:8749. doi: 10.3390/ijerph18168749, 34444504 PMC8394549

[3] Torossian A Bräuer A Höcker J Bein B Wulf H Horn EP. Preventing inadvertent perioperative hypothermia. Dtsch Arztebl Int. (2015) 112:166–72. doi: 10.3238/arztebl.2015.0166, 25837741 PMC4383851

[4] Wongyingsinn M Pookprayoon V. Incidence and associated factors of perioperative hypothermia in adult patients at a university-based, tertiary care hospital in Thailand. BMC Anesthesiol. (2023) 23:137. doi: 10.1186/s12871-023-02084-2, 37098492 PMC10127435

[5] Kurz A Sessler DI Lenhardt R. Perioperative normothermia to reduce the incidence of surgical-wound infection and shorten hospitalization. Study of wound infection and temperature group. N Engl J Med. (1996) 334:1209–16. doi: 10.1056/NEJM199605093341901, 8606715

[6] Schmied H Kurz A Sessler DI Kozek S Reiter A. Mild hypothermia increases blood loss and transfusion requirements during total hip arthroplasty. Lancet. (1996) 347:289–92. doi: 10.1016/s0140-6736(96)90466-3, 8569362

[7] Dean M Ramsay R Heriot A Mackay J Hiscock R Lynch AC. Warmed, humidified CO_2_ insufflation benefits intraoperative core temperature during laparoscopic surgery: a meta-analysis. Asian J Endosc Surg. (2017) 10:128–36. doi: 10.1111/ases.12350, 27976517 PMC5484286

[8] Sessler DI. Temperature monitoring and perioperative thermoregulation. Anesthesiology. (2008) 109:318–38. doi: 10.1097/ALN.0b013e31817f6d76, 18648241 PMC2614355

[9] Simegn GD Bayable SD Fetene MB. Prevention and management of perioperative hypothermia in adult elective surgical patients: a systematic review. Ann Med Surg. (2021) 72:103059. doi: 10.1016/j.amsu.2021.103059, 34840773 PMC8605381

[10] Campbell G Alderson P Smith AF Warttig S. Warming of intravenous and irrigation fluids for preventing inadvertent perioperative hypothermia. Cochrane Database Syst Rev. (2015) 2015:CD009891. doi: 10.1002/14651858.CD009891.pub2, 25866139 PMC6769178

[11] Yi J Xiang Z Deng X Fan T Fu R Geng W . Incidence of inadvertent intraoperative hypothermia and its risk factors in patients undergoing general anesthesia in Beijing: a prospective regional survey. PLoS One. (2015) 10:e0136136. doi: 10.1371/journal.pone.0136136, 26360773 PMC4567074

[12] Chen HY Su LJ Wu HZ Zou H Yang R Zhu YX. Risk factors for inadvertent intraoperative hypothermia in patients undergoing laparoscopic surgery: a prospective cohort study. PLoS One. (2021) 16:e0257816. doi: 10.1371/journal.pone.0257816, 34555101 PMC8460038

[13] Sari S Aksoy SM But A. The incidence of inadvertent perioperative hypothermia in patients undergoing general anesthesia and an examination of risk factors. Int J Clin Pract. (2021) 75:e14103. doi: 10.1111/ijcp.14103, 33616248

[14] Hu Z Li W Liang C Li K. Risk factors and prediction model for inadvertent intraoperative hypothermia in patients undergoing robotic surgery: a retrospective analysis. Sci Rep. (2023) 13:3687. doi: 10.1038/s41598-023-30819-1, 36878972 PMC9988985

[15] Cao B Li Y Liu Y Chen X Liu Y Li Y . A multi-center study to predict the risk of intraoperative hypothermia in gynecological surgery patients using preoperative variables. Gynecol Oncol. (2024) 185:156–64. doi: 10.1016/j.ygyno.2024.02.009, 38428331

[16] Yan L Yao L Zhao Q Xiao M Li Y Min S. Risk prediction models for inadvertent intraoperative hypothermia: a systematic review. J Perianesth Nurs. (2021) 36:724–9. doi: 10.1016/j.jopan.2021.02.011, 34663532

[17] Collins GS Reitsma JB Altman DG Moons KG. Transparent reporting of a multivariable prediction model for individual prognosis or diagnosis (TRIPOD): the TRIPOD statement. BMJ. (2015) 350:g7594. doi: 10.1136/bmj.g7594, 25569120

[18] Collins GS Moons KGM Dhiman P Riley RD Beam AL van Calster B . TRIPOD+AI statement: updated guidance for reporting clinical prediction models that use regression or machine learning methods. BMJ. (2024) 385:e078378. doi: 10.1136/bmj-2023-078378, 38626948 PMC11019967

[19] Balachandran VP Gonen M Smith JJ DeMatteo RP. Nomograms in oncology: more than meets the eye. Lancet Oncol. (2015) 16:e173–80. doi: 10.1016/S1470-2045(14)71116-7, 25846097 PMC4465353

[20] Iasonos A Schrag D Raj GV Panageas KS. How to build and interpret a nomogram for cancer prognosis. J Clin Oncol. (2008) 26:1364–70. doi: 10.1200/JCO.2007.12.9791, 18323559

[21] Riley RD Ensor J Snell KIE Harrell FE Jr Martin GP Reitsma JB . Calculating the sample size required for developing a clinical prediction model. BMJ. (2020) 368:m441. doi: 10.1136/bmj.m441, 32188600

[22] Frank SM Raja SN Bulcao C Goldstein DS. Age-related thermoregulatory differences during core cooling in humans. Am J Physiol Regul Integr Comp Physiol. (2000) 279:R349–54. doi: 10.1152/ajpregu.2000.279.1.R349, 10896899

[23] Sessler DI. Perioperative heat balance. Anesthesiology. (2000) 92:578–96. doi: 10.1097/00000542-200002000-00042, 10691247

[24] Yi J Lei Y Xu S Si Y Li S Xia Z . Intraoperative hypothermia and its clinical outcomes in patients undergoing general anesthesia: national study in China. PLoS One. (2017) 12:e0177221. doi: 10.1371/journal.pone.0177221, 28594825 PMC5464536

[25] Riley C Andrzejowski J. Inadvertent perioperative hypothermia. BJA Educ. (2018) 18:227–33. doi: 10.1016/j.bjae.2018.05.003, 33456837 PMC7807998

[26] Rajagopalan S Mascha E Na J Sessler DI. The effects of mild perioperative hypothermia on blood loss and transfusion requirement. Anesthesiology. (2008) 108:71–7. doi: 10.1097/01.anes.0000296719.73450.52, 18156884

[27] Cheng Y Sheng H. Risk factors and predictive modeling of intraoperative hypothermia in laparoscopic surgery patients. BMC Surg. (2025) 25:426. doi: 10.1186/s12893-025-03186-z, 41044570 PMC12495671

[28] Yi J Zhan L Lei Y Xu S Si Y Li S . Establishment and validation of a prediction equation to estimate risk of intraoperative hypothermia in patients receiving general anesthesia. Sci Rep. (2017) 7:13927. doi: 10.1038/s41598-017-12997-x, 29066717 PMC5654776

[29] Bessell JR Karatassas A Patterson JR Jamieson GG Maddern GJ. Hypothermia induced by laparoscopic insufflation. A randomized study in a pig model. Surg Endosc. (1995) 9:791–6. doi: 10.1007/BF00190083, 7482186

[30] Yan L Tan J Chen H Xiao H Zhang Y Yao Q . A nomogram for predicting unplanned intraoperative hypothermia in patients with colorectal Cancer undergoing laparoscopic colorectal procedures. AORN J. (2023) 117:e1–e12. doi: 10.1002/aorn.13845, 36573748

[31] Shen C He Y. Intraoperative hypothermia in patients with laparoscopic surgery: influencing factors and prevention strategies. Heliyon. (2024) 10:e31479. doi: 10.1016/j.heliyon.2024.e31479, 38831829 PMC11145475

[32] Liu J Liu F Xu W du L Li Y Liang A . Risk prediction models for perioperative hypothermia: a systematic review. J Multidiscip Healthc. (2025) 18:4443–52. doi: 10.2147/JMDH.S538891, 40756615 PMC12314651

