# Supplementary figures and images for: Development and internal validation of a nomogram to predict perioperative hypothermia in patients undergoing laparoscopic gynecologic surgery under general anesthesia: a retrospective cohort study
Source: Front Med (Lausanne). 2026 Jun 10;13:1799286. doi: 10.3389/fmed.2026.1799286 (PMC13290850; doi:10.3389/fmed.2026.1799286)

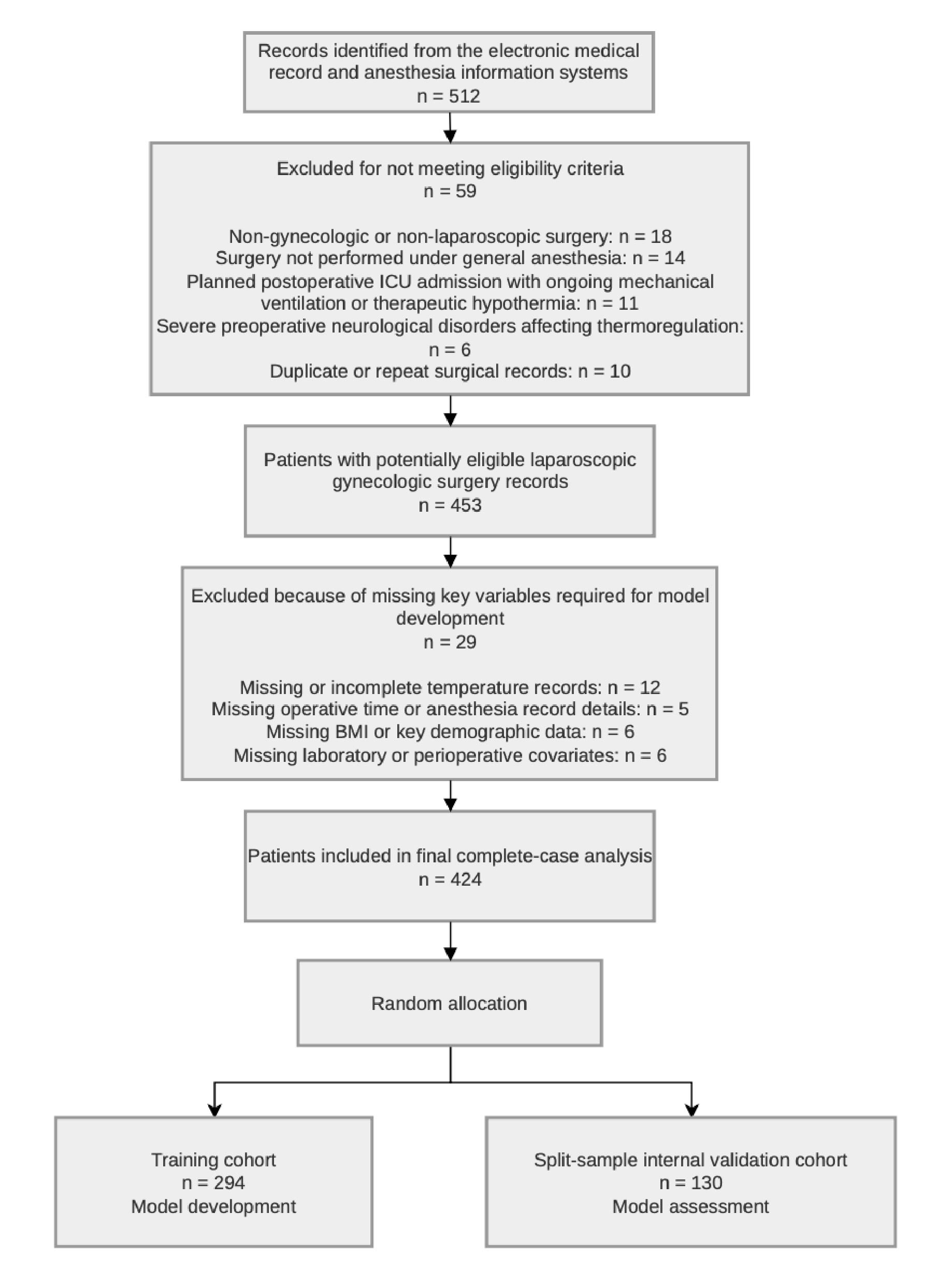

Supplement: Supplementary file 1 [file Image_1.JPEG]
