# Supplementary material for: Development and internal validation of a nomogram to predict perioperative hypothermia in patients undergoing laparoscopic gynecologic surgery under general anesthesia: a retrospective cohort study
Source: Front Med (Lausanne). 2026 Jun 10;13:1799286. doi: 10.3389/fmed.2026.1799286 (PMC13290850; doi:10.3389/fmed.2026.1799286)
Supplement: Supplementary file 2 [file Table_1.DOCX]

**Supplementary Table S1.** Completed TRIPOD/TRIPOD+AI checklist for reporting the present prediction model study.

| **Item/domain** | **Reporting element** | **Location in revised manuscript** | **Status/comment** |
| --- | --- | --- | --- |
| Title | Identification as a prediction model development and internal validation study | Title | Reported |
| Abstract | Study design, setting, population, outcome, model development, internal validation, and performance | Abstract | Reported |
| Background | Clinical context and rationale for prediction model development | Introduction | Reported |
| Intended use | Intended use of the nomogram for risk stratification, active warming triage, and quality-improvement audits | Introduction; Discussion | Added in revision |
| Source of data | Study setting, time period, and retrospective data source | Methods: Setting; Data sources and measurement | Reported |
| Participants | Eligibility criteria, restriction to laparoscopic gynecologic surgery, and exclusion criteria | Methods: Participants | Reported |
| Outcome | Definition of perioperative hypothermia as core temperature <36.0 °C during intraoperative or immediate postoperative period | Methods: Outcome | Reported |
| Candidate predictors | Demographic, clinical, intraoperative, and management variables selected a priori | Methods: Predictors and covariates | Reported |
| Sample size | Available-data sample size, event number, and EPV-based assessment | Methods: Study size | Reported; further limitations acknowledged |
| Missing data | Complete-case analysis and no imputation | Methods: Statistical analysis | Reported |
| Model development | LASSO regression, Boruta algorithm, and final logistic regression model | Methods: Statistical analysis; Results | Reported |
| Model specification | Final model predictors and nomogram presentation | Results; Figure 2 | Reported |
| Performance measures | AUC, calibration plots, and decision curve analysis | Methods; Results; Figures 3–4 | Reported |
| Internal validation | Split-sample internal validation cohort | Methods; Results | Reported |
| Interpretation | Scope, intended use, comparison with existing models, and limitations | Discussion | Reported and expanded |
| Transparency | Completed checklist provided as supplementary material | Supplementary Table S1 | Added in revision |

**Supplementary Table S2.** Bootstrap optimism-corrected internal validation of the final model

| **Metric** | **Apparent performance** | **Optimism-corrected performance** |
| --- | --- | --- |
| AUC | 0.794 | 0.776 |
| Calibration slope | 1.00 | 0.91 |
| Brier score | 0.189 | 0.197 |

Note: The final model included age, body mass index, and operative time. Bootstrap optimism-corrected internal validation was performed using 500 resamples in the full cohort. Apparent performance refers to performance in the original dataset, whereas optimism-corrected performance accounts for bootstrap-estimated optimism. AUC, area under the receiver operating characteristic curve. A calibration slope closer to 1.00 and a lower Brier score indicate better model performance.

**Supplementary Table S3.** Non-zero LASSO coefficients at λmin

| **Variable** | **Coefficient** |
| --- | --- |
| Age | 0.043 |
| Body mass index | -0.118 |
| Operative time | 0.005 |
| Active warming used | -0.356 |
| ASA class III vs I | 0.214 |

Note: LASSO regression was performed using cv.glmnet with family = "binomial" and ten-fold cross-validation in the training cohort. Continuous variables were standardized during model fitting, and categorical variables were encoded as dummy variables. Coefficients shown are non-zero coefficients at λ_min. Active warming was retained in the LASSO screening step but was prespecified as ineligible for the final clinical nomogram because it represented a treatment-related, decision-dependent variable.

**Supplementary Table S4.** Calibration metrics of the final prediction model

| **Metric** | **Training cohort** | **Validation cohort** |
| --- | --- | --- |
| Calibration intercept | 0.01 | −0.04 |
| Calibration slope | 0.98 | 0.93 |
| Integrated calibration index | 0.035 | 0.046 |
| Expected-to-observed ratio | 1.00 | 0.98 |
| Brier score | 0.192 | 0.187 |
| Hosmer–Lemeshow χ² | 6.84 | 7.31 |
| Hosmer–Lemeshow P value | 0.554 | 0.503 |

Note: The Hosmer–Lemeshow test was used as an additional numerical goodness-of-fit assessment. A non-significant P value indicates no statistical evidence of poor calibration. Calibration results should be interpreted together with calibration plots, calibration slope, calibration intercept, ICI, E/O ratio, and Brier score.

**Supplementary Table S5. Regression coefficients of the final three-predictor model**

| **Predictor** | **Coefficient β** | **Standard error** | **Odds ratio** | **95% CI** | **P value** |
| --- | --- | --- | --- | --- | --- |
| Intercept | −2.684 | 0.612 | — | — | <0.001 |
| Age, per year | 0.061 | 0.011 | 1.063 | 1.041–1.086 | <0.001 |
| BMI, per kg/m² | −0.184 | 0.032 | 0.832 | 0.781–0.886 | <0.001 |
| Operative time, per minute | 0.006 | 0.001 | 1.006 | 1.004–1.009 | <0.001 |

Note: The final prediction model was fitted in the training cohort using logistic regression and included only the three predictors retained after LASSO/Boruta feature selection and prespecified conceptual review. The predicted probability of perioperative hypothermia can be calculated as:

p = 1 / [1 + exp − (−2.684 + 0.061 × age − 0.184 × BMI + 0.006 × operative time)].

BMI, body mass index; CI, confidence interval.
